# EVALUATION OF CLINICOMYCOLOGICAL ASPECTS OF ONYCHOMYCOSIS

**DOI:** 10.4103/0019-5154.44788

**Published:** 2008

**Authors:** Ravinder Kaur, Bineeta Kashyap, Rati Makkar

**Affiliations:** *From the Department of Microbiology, Maulana Azad Medical College, Bahadur Shah Zafar Marg, New Delhi - 110 002, India*; 1 *From the Department of Dermatology, Maulana Azad Medical College, Bahadur Shah Zafar Marg, New Delhi - 110 002, India*

**Keywords:** *Culture*, *diagnosis*, *drilling*, *nails*, *onychomycosis*

## Abstract

**Background:**

Incidence of onychomycosis has increased tremendously in recent times. Relatively little work has been done on this problem in our country. Research in past has been concentrated mainly on superficial mycoses of the skin.

**Aim:**

It is a well-established fact that geographical distribution of the fungi may change from time to time; hence, this study was planned to analyze clinical, epidemiological, and mycological features of onychomycosis.

**Materials and Methods:**

Sixty patients clinically suspected and microscopically proven to have onychomycosis were taken up for the study. Nail samples, collected by scraping in 30 patients and by using a dental drill in the rest, were examined microscopically and cultured for fungus.

**Results and Conclusions:**

Forty-five fungal isolates were obtained from 60 patients. Trichophyton rubrum was the commonest fungus isolated (46.67%). Trichophyton mentagrophytes and Candida albicans accounted for 20% and 15.56% isolates, respectively. Two cases showed mixed growth of Trichophyton rubrum and Aspergillus niger in one and Trichophyton rubrum and Aspergillus fumigatus in the other. Isolation rate was higher by drilling compared to scraping, the rates being 83.33% and 66.67%, respectively. Superficial mycotic infections were present in 27 patients (45%).

## Introduction

Fungal infections of the nails also called onychomycosis are commonest nail disorder encountered in clinical practice constituting 20%−40% of all diseases of nails^1^ and 30% of superficial mycotic infections.^2^ Various Indian workers have reported incidence to be at 0.5%−5% in the general population.^3^,^4^ Compared to the other superficial mycoses, this condition is persistent, intractable and poses serious concern to the clinicians as it often becomes a chronic source of recurrent superficial mycotic skin infections, besides causing considerable disfigurement.^5^,^6^

Dermatophytes cause 90% of toenails and 50% of fingernail onychomycosis.^7^ Candida species, particularly *Candida albicans*, prevail in fingernail infections.^8^ Nondermatophytic molds are rare, although few species are described as etiological agents of onychomycosis.^9^

Combination of time-honored techniques of KOH mount and culture form the gold standard for the diagnosis of onychomycosis.^10^ Positivity by KOH mount is between 40% and 65% that could be due to improper technique or presence of scanty material.^9^ Yield of the fungal cultures is reported to be 50%–75% in KOH positive specimens and use of dental drill raises the success rate of cultures of KOH positive nails.^11^

Relatively little work has been done on this problem in our country. Further, it is a well-established fact that geographical distribution of the fungi may change from time to time; hence, this study was planned to analyze the clinical, epidemiological, and mycological features of onychomycosis.

## Materials and Methods

Sixty patients clinically suspected and microscopically proven with 20% KOH to have onychomycosis were taken up for the study. Clinical history was taken and a thorough examination of the patients was conducted. Presence of any associated skin or systemic disease was recorded.

In 30 patients, nail samples were collected by scraping till the junction of the healthy and the diseased nail, including the subungual debris from under the distal edge of nails avoiding any discomfort to the patients. A dental drill was used to collect the nail dust in the remaining 30 patients. In cases where both finger and the toenails were involved, samples were taken from both. The scrapings were also taken from associated fungal lesions of the skin if any from the outer edge of the lesions.

For culture, all the samples were inoculated into two sets of media-Sabouraud's dextrose agar with chloramphenicol and Sabouraud's dextrose agar with chloramphenicol and cycloheximide. Both were incubated at 25°C and 37° and examined for growth for six weeks; after which if no growth was observed, the sample was reported as negative. In most cases, the culture was repeated more than once and in cases where contamination was suspected subculture was done. The identification of the isolates was done in accordance with standard recommended techniques.^12^

## Results

### Demographic profile

**Fig. 1:**
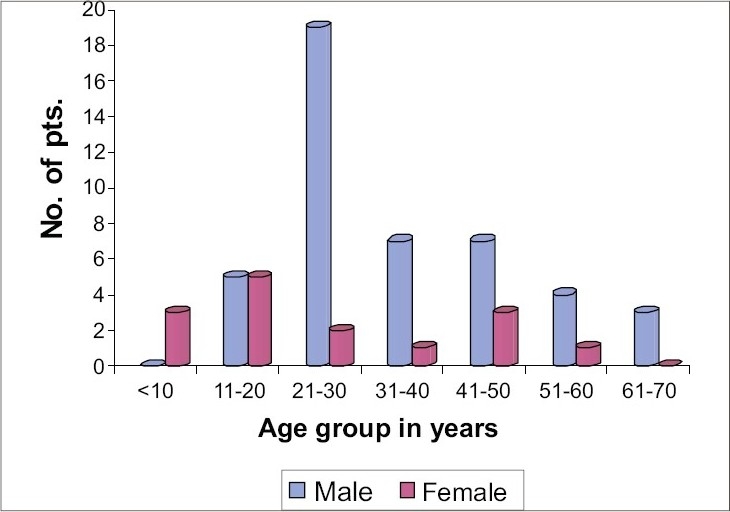
Distribution of cases in relation to age and sex

In the present study, onychomycosis was seen to affect all ages ranging from 5 years to 67 years, the mean age being 31.72 years and the majority of cases were males (Fig. 1). As many as 51 patients (85%) were living in urban areas, while only 9 (15%) came from rural areas. One patient (1.67%) was in the professional group and others were more or less equally distributed in other groups (i.e., housewife, agriculture, laborer, industrial worker, clerical, students, others). Majority were matriculate or more qualified constituting 71.93% of total cases and only 16 patients (28.07%) were illiterate (3 patients <10 years not included). Most patients were involved in domestic activities (33.33%), the most common being cooking followed by stitching and tailoring. Only 1 patient (1.67%) was fond of playing outdoor games.

### Clinical aspects

**Fig. 2:**
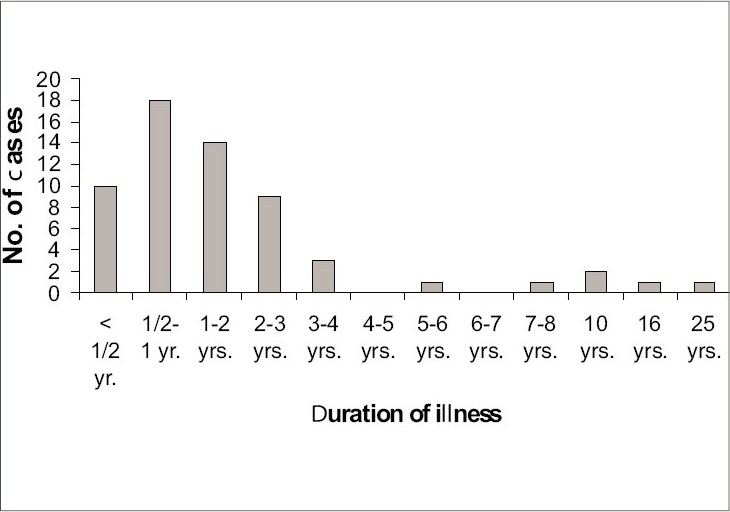
Distribution of cases in relation to duration of illness

**Fig. 3:**
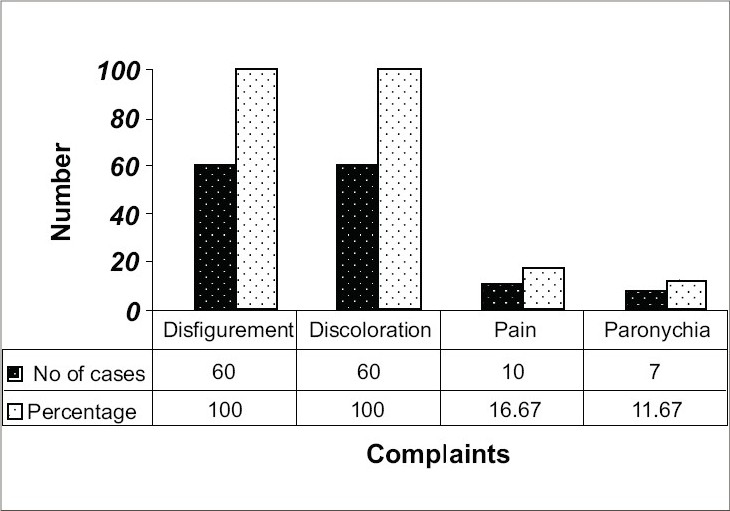
Frequency of presenting complaints

Fig. 2 shows distribution of cases in relation to duration of illness. The frequency of the presenting complaints is shown in Fig. 3. There was history of trauma preceding nail involvement in 3 (5%) patients. 59 of 60 cases (98.33%) cut their nails themselves, while in only 1 patient (1.67%) belonging to rural area, the nails were pared by a barber. One patient (1.67%) gave history of onychophagia. Family history of fungal infection of nails could be elicited in 4 (6.67%) of 60 patients. History of contact with the cattle and pets was present in 11 patients (18.67%). Of the total patients, 7 (11.67%) had taken treatment with griseofulvin for 3–4 months, while 2 (3.33%) received homeopathic therapy. Regarding the footwear habits of patients, 29 (48.33%) were in the habit of wearing chappals, 20 (33.33%) of them used to wear socks and shoes for most part of the day, 5 (8.33%) used to wear only shoes and only 6 (10%) were in the habit of walking barefoot.

### Examination

**Fig. 4:**
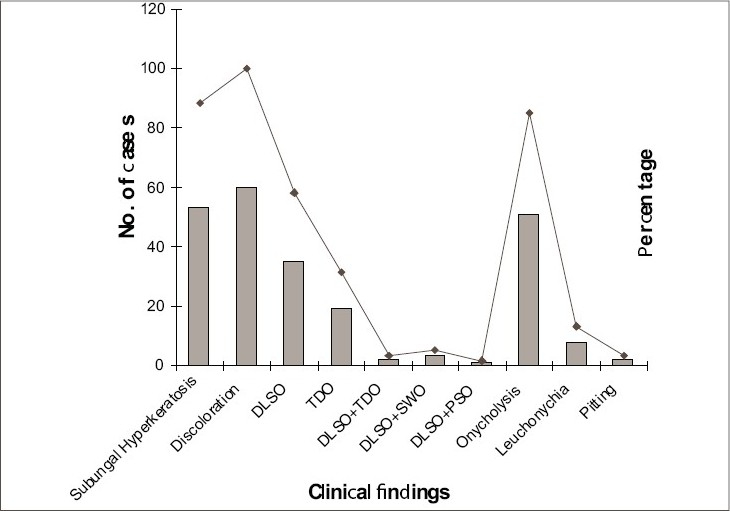
Nail plate changes in patients with onychomycosis

Out of 60 patients, 36 (60%) showed involvement of hands among whom 14, 9 and 13 had involvement of right, left and both hands, respectively, whereas 11 (18.33%) patients had involvement of feet with 1, 1 and 9 in right, left and both feet, respectively. It was observed that patients who were in the habit of wearing chappals had much less incidence of toenail involvement (2/29) as compared to patients used to wearing shoes and socks (14/20), shoes only (4/5) or barefoot (4/6). Onychomycosis was limited to only one nail in 10/60 (16.67%) cases, while 50 patients (83.33%) showed involvement of two or more nails. The most common findings seen on examination of nails were subungual hyperkeratosis and discoloration (Fig. 4). Presence of coexisting fungal infections in other parts of the body was noted in 27 (45%) patients, the most frequent being *Tinea manum*. Other skin/systemic disorder was present in 23 (38.33%) patients, hyperhidrosis being the most frequent.

### KOH mounts and culture

Rate of isolation of fungus was higher in nail samples collected by drilling (83.33%) than scraping (66.67%), and this difference was statistically significant using the Chi-Square test. Fungi isolated were *T. rubrum* in 21 (46.67%), *T. mentagrophytes* in 9 (20%), *C. albicans* in 7 (15.56%), *T. tonsurans* and *A. niger* in 2 (4.44%) each and *T. violaceum*, *T.schoenleinii*, *T. rubrum + A. niger* and *T. rubrum + A. fumigatus* in 1 (2.22%) case each. Fungi isolated from fingernails only included *T. rubrum*, *T. mentagrophytes*, *T. violaceum*, *C. albicans* and Aspergillus species with *T. rubrum* and *C. albicans* in 16 and 7 cases, respectively. Common fungi isolated from toenails only were *T. rubrum*, *T. mentagrophytes*, *T. tonsurans* and *A. niger* with *T. mentagrophytes* in 4 cases, whereas those isolated from both finger and toe nails simultaneously were *T. rubrum*, *T. mentagrophytes*, *T. violaceum*, *T. tonsurans* and *T. schoenleinii* with *T. rubrum* and *T. mentagrophytes* in 4 cases each, respectively. Of the 27 skin samples subjected to fungul culture, 14 (51.85%) showed growth of the same fungus as isolated from nails. With regard to the type of nail dystrophy in relation to the causative fungus, *Trichophyton rubrum* (*T. rubrum)* was the commonest isolate obtained from cases of DLSO (distal and lateral subungual onychomycosis) and TDO (total dystrophic onychomycosis) and the only isolate obtained from PSO (proximal subungual onychomycosis) was *T. rubrum*. *Trichophyton mentagrophytes* (was isolated from 2 cases of SWO (superficial white onychomycosis) and *A. niger* from the third case. Superficial fungal skin infection was present in 15 of the 21 (71.4%) *T. rubrum* positive cases and in 5 of the 9 (55.55%) *T. mentagrophytes* cases. Of the two cases with mixed infections, one had associated skin infection and none of the other dermatophytes, *A. niger* or *C. albicans,* were associated with any such infection. Samples from only 41 patients were subjected to periodic acid schiff (PAS) staining, the rest being inadequate, and out of these 21 (51.22%) were reported PAS positive.

Forty-five fungal isolates were obtained from 60 patients. Trichophyton rubrum was the commonest fungus isolated (46.67%). Trichophyton mentagrophytes and Candida albicans accounted for 20% and 15.56% isolates, respectively. Two cases showed mixed growth of Trichophyton rubrum and Aspergillus niger in one and Trichophyton rubrum and Aspergillus fumigatus in the other. Isolation rate was higher by drilling compared to scraping, the rates being 83.33% and 66.67%, respectively. Superficial mycotic infections were present in 27 patients (45%).

## Discussion

Age distribution of onychomycosis in our study population is consistent with the view that onychomycosis is disease of adults and is uncommon in children except where the patient or family members are suffering from superficial fungal infection.^13^ In our case, 1 out of the 3 children (33.33%) had concurrent *Tinea capitis.* The highest and lowest incidence in 21–30 years and 61–70 years age group, respectively, are similar to those in earlier studies.^13^ The prevalence in elderly in our study is lower than the reported figures,^14^ which could be due to lower presentation rate to the hospital. Onychomycosis was found to be more common in males than females similar to the observation reported by most of the workers.^3^,^15^ Few isolated reports of female preponderance are also present in literature.^14^ Lesser incidence of onychomycosis in females as compared to males may be more apparent than real because of underreporting.^15^

Candidal onychomycosis, being symptomatic has been reported to be more common in females by almost all workers similar to ours.^14^,^16^ Most patients were involved in occupation that predispose to repeated minor trauma and all the seven patients of candidal onychomycosis were engaged in domestic activities that involved wet work, suggesting that healthy nail cannot be infected by fungus as reported by many other workers.^15^,^17^ Infections caused by dermatophytes and moulds were largely asymptomatic as suggested by late presentation to the hospital (30% between 6 months and 1 year), whereas those by candida were all associated with discoloration, disfigurement, pain, swelling and redness and sought treatment earlier, as reported earlier.^6^,^14^–^16^ Earlier workers have emphasized importance of trauma preceding infection though only 3 patients in our study gave history of trauma.^17^ Our study shows that occlusion, warmth and moisture provided by occlusive footwear predispose to onychomycosis as reported earlier.^17^ Onychomycosis that occurred in 4 of the 6 patients barefoot could be explained by their occupational predisposition to trauma (farmer/laborer).

Our study shows that fingernails alone are involved most commonly in onychomycosis as shown by other Indian workers,^15^ unlike western studies^17^,^18^ that could be explained by widespread adoption of occlusive footwear in western countries and much lower level of cosmetic consciousness in our people resulting in lower reporting of toenail infections. Right hand was more commonly involved, as microtrauma is more common in the hand used most. Subungual hyperkeratosis, most reliable sign of onychomycosis, was present in 88.33% cases and discoloration, earliest feature of fungal infection, was present in all cases irrespective of paronychia as reported by others.^19^ Our finding of onycholysis in all, except early cases, is consistent with earlier findings.^17^ Once fungus is established in nails, infected nails act as reservoir of organism providing a constant source of infection for other parts of body,^6^,^19^ as suggested by the presence of concurrent fungal infection in 27 out of 60 (45%) cases with onychomycosis. As has been reported by Hay *et al.*,^20^ no correlation was found in diabetes mellitus and fungal infection of nails.

In our study, rate of isolation increased to 83.33% by drilling from 66.67%. Difficulty in isolating fungi from nail clippings in cases of onychomycosis because of nonviability of the fungal hyphae in distal portion of nail plate from where scraping is done is well known^15^,^17^ and an increase in positivity rate to 88% from the usually reported rates of 50%–75% have been reported by use of drill which allows sampling from the proximal end.^21^ It is a well-established fact that geographical distribution of the fungi may change from time to time. Dermatophytes are the most frequently implicated causative agents in onychomycosis, whereas yeasts, which were previously regarded as contaminants, are now increasingly recognized as pathogens in fingernail infections, as are some moulds.^22^ The incidence and clinical significance of other than dermatophytic fungi or molds causing onychomycosis is not well known because they may be colonizing organisms rather than pathogens; however, several reports have described a number of species such as Fusarium species, Scytalidium species, and Acremonium species as etiological agents of onychomycosis.^23^ Since this article is a small article, it is not possible to present an elaborate description of these agents; however, one review article of ours that describes the isolation rates of different fungi from different regions is already in press.^24^ Dermatophytes were responsible for 80% of the total cases of onychomycosis in the present study, the most common isolate being *T. rubrum* (46.67%) as has been established by previous studies.^17^,^19^,^20^ Nondermatophytes in our study formed 4.44% of the total isolates and included Aspergillus species only that has been earlier reported from India^25^. The prevalence of molds in onychomycosis is reported between 1.5%–6% by various authors.^2^ Our study shows 2 cases of mixed infection which suggests that mixed infections are described but are uncommon.^17^,^26^ *T. rubrum* has been reported to be the commonest in fingernail infection as in our study,^2^,^26^ due to better adaptation, more virulence and easy colonization on hard keratin. *C. albicans* was isolated in 7 cases of fingernail infection, all having paronychia which is in agreement with various studies reporting that candida is almost exclusively found in fingernails in conjunction with candidal paronychia.^15^,^17^ Although *T. mentagrophytes* is reported to be more common in the toenails,^17^ *T. rubrum* is still more common than *T. mentagrophytes* in toenails as shown by various studies.^20^,^26^ 71.4% cases caused by *T. rubrum* and 55.55% cases caused by *T. mentagrophytes* were associated with skin infections whereas *C. albicans* and *A. niger* has no such association. These findings are in agreement with the previous observations^20^ that *T. rubrum* spreads readily to all the parts of body as compared to *T. mentagrophytes* and onychomycosis due to saprophytic moulds is not accompanied by superficial mycotic infection. Histopathology, which showed PAS positivity of 51.22% in our case, has been shown to be a simple, rapid and sensitive technique for diagnosis of fungal infection resulting in PAS positivity from 41.3% to 60%.^11^,^27^
